# Modulation of chemoimmunotherapy efficacy in non-small cell lung cancer by sex and histology: a real-world, patient-level analysis

**DOI:** 10.1186/s12885-022-09187-y

**Published:** 2022-01-19

**Authors:** Stephanie Tuminello, Naomi Alpert, Rajwanth R. Veluswamy, Arvind Kumar, Jorge E. Gomez, Raja Flores, Emanuela Taioli

**Affiliations:** 1 Institute for Translational Epidemiology and Department of Population Health Science and Policy, Icahn School of Medicine at Mount Sinai, One Gustave L. Levy Place, Box 1133, New York, NY 10029 USA; 2 Department of Population Health, New York University Langone Health, New York, NY 10016 USA; 3 Department of Medicine, Hematology and Medical Oncology, Icahn School of Medicine at Mount Sinai, New York, NY USA; 4 Tisch Cancer Institute, Icahn School of Medicine at Mount Sinai, New York, NY USA; 5 The Hasso Plattner Institute for Digital Health at Mount Sinai, Icahn School of Medicine at Mount Sinai, New York, NY USA; 6 Department of Thoracic Surgery, Icahn School of Medicine at Mount Sinai, New York, NY USA

**Keywords:** Immunotherapy, NSCLC, Immune checkpoint inhibitors, Squamous cell carcinoma

## Abstract

**Background:**

It has been postulated that patient’s sex impacts response to immunotherapy. Sex modulation of immunotherapy benefit, however, has not yet been explored using patient-level data, where potential confounders, as well as histologic type, can be accounted for. Here we investigated the association between sex and chemoimmunotherapy efficacy for non-small cell lung cancer (NSCLC) using a large, nation-wide dataset.

**Patients & methods:**

Stage IV NSCLC patients diagnosed in 2015 were identified in the National Cancer Database (NCDB). Patients were treated with either chemoimmunotherapy or chemotherapy alone. The efficacy of the addition of immunotherapy treatment by sex was investigated using both an adjusted Cox proportional hazards model and propensity-score matching, in both the overall cohort and stratified by histological subtype.

**Results:**

2064 (16%) patients received chemoimmunotherapy and10,733 (84%) received chemotherapy alone. Adjusted survival analysis in the overall cohort showed that both males (hazards ratio (HR)_adj_: 0.80, 95% CI: 0.74–0.87) and females (HR_adj_: 0.83, 95% CI: 0.76–0.90) had better OS when treated with chemoimmunotherapy than chemotherapy alone, with no statistically significant interaction between sex and receipt of immunotherapy (*p* = 0.63). Propensity matching confirmed these results. However, for those with squamous cell histology, male patients derived more benefit from chemoimmunotherapy treatment than females (HR_adj_: 0.73, 95% CI: 0.58–0.91 vs HR_adj_: 1.03, 95% CI: 0.76–1.38; p for interaction = 0.07).

**Conclusion:**

Male patients with squamous cell carcinoma may derive more benefit from chemoimmunotherapy treatment. Histology likely plays an important role in how sex modulates immunotherapy efficacy.

**Supplementary Information:**

The online version contains supplementary material available at 10.1186/s12885-022-09187-y.

## Introduction

Platinum-based doublet chemotherapy was the mainstay treatment for advanced, non-small cell lung cancer (NSCLC) for decades, until, in 2015, the FDA approved Pembrolizumab for patients with metastatic disease who had: 1) failed to respond to other treatments and 2) had tumors expressing PD-L1 [1]. Immune checkpoint inhibitors (either anti-CTLA-4 or anti-PD-1/PD-L [Pembrolizumab]) work by allowing T-lymphocytes to overcome checkpoints imposed by tumor cells, thus galvanizing the anti-tumor immune response [2]. Advanced NSCLC patients treated through immune-checkpoint inhibition show significantly improved survival rates [3–5]. While treatment paradigms continue to evolve, most patients with advanced-stage NSCLC without targetable driver mutations receive a combination of immunotherapy and chemotherapy as front-line treatment.

However, not all NSCLC patients experience benefit from immunotherapy, suggesting factors modulate immunotherapy response, with clinical characteristics acting as surrogates for underlying biological processes [6]. Sex, specifically, may play an important role in immunotherapy efficacy. Females in general are known to mount stronger innate and adaptive immune responses, as evidenced by their: 1) ability to clear pathogens faster; 2) greater antibody production in response to vaccinations; and 3) higher rates of autoimmune disorders compared to males [7–10]. A stronger anti-tumor immune response in females may be responsible for the improved cancer-related outcomes seen across several cancer types and stages. Moreover, experimental evidence from animal models suggests sex-hormone modulation of the PD-1–PD-L1 pathway of immune regulation [11, 12]. Conversely, cancers in males may have higher immunogenicity as a result of sex-related differences in mutagenic risk factors, mainly smoking and occupational exposure [13].

A recent meta-analysis of randomized clinical trials (RCTs) suggested that male patients with advanced solid malignancies derive more benefit from immune checkpoint inhibitors than females [12]. However, the same group reported the converse to be true when analyzing the effect of the combination of PD-L1/PD-1 inhibitors and chemotherapy, with female patients experiencing a greater degree of benefit [14]. These results imply that any postulated sex-based differences in immunotherapy response result from complex interactions of underlying biological and environmental factors. However, these and other prior analyses exploring the role of sex and immunotherapy response have not accounted for key patient-level data [12, 15] such as age, comorbidity, and histologic type, which may be modifying the relationship between patients’ sex and immunotherapy efficacy.

In this study we used hospital-based, individual-level data to investigate whether the efficacy of chemoimmunotherapy compared to chemotherapy alone differs according to sex in stage IV NSCLC patients, adjusting for key clinical covariates. Because histology is an important factor in treatment decision, safety and efficacy in NSCLC, [16] we also stratified by histological type. As a secondary aim, we limited to the subset of NSCLC patients treated with chemoimmunotherapy to assess which sex, on average, had better survival outcomes.

## Methods

### Study population

The study cohort was identified from the National Cancer Database (NCDB), a joint project between the American College of Surgeons and the American Cancer Society, which provides clinical information for over 70% of annual incident cancer diagnoses nationwide [17]. We included all patients with pathologically confirmed, primary stage IV NSCLC (American Joint Committee on Cancer staging manual, 7th edition), who were diagnosed in 2015, and treated with either chemotherapy alone or combination of chemotherapy with immunotherapy as first line treatment. 2015 was the most recent year of NCDB data available to us with sufficient follow-up information for survival analysis.

Patients without complete treatment information were excluded. Patients were also excluded if they received doses of radiotherapy greater than what would be given for palliative care (> 4000 Gy) or if radiation dosage was unknown. Patients with a recorded brain metastasis at the time of diagnosis and subsequent brain radiation were also excluded due to the historically worse outcomes these patients experience regardless of systemic treatment choice. Lastly, patients who had a contraindication to immunotherapy or had missing data to confirm whether they received immunotherapy as first line were also excluded (Supplemental Fig. 1).

### Variables

From the NCDB we extracted information on patient’s sex, age at diagnosis, race (white, Black or Asian/other), Charlson-Deyo comorbidity index score, tumor histology (adenocarcinoma, squamous or large cell/other), receipt of palliative radiation, insurance status (uninsured, private, public or unknown), median household income for the patient area of residence and year of diagnosis. NCDB provides first line cancer-directed treatment, which we used to classify patients as receiving either chemotherapy alone, or chemoimmunotherapy. Receipt of immunotherapy was specifically determined by using the NCDB variable RX_SUMM_IMMUNOTHERAPY, which is defined as receiving “biological or chemical agents that alter the immune system or change the host’s response to tumor cells” [17, 18]. However, the specific type of immunotherapy received by each study patient is not provided. Agents included in the NCDB immunotherapy variable include immune checkpoint inhibitors (e.g., anti-PD1/PDL1 or anti-CTLA4 inhibitors), tumor vaccines, and other immunomodulatory drugs (Supplemental Table 1) [17–19]; many of these agents are described in the code book as being used as part of clinical trials. A complete list of agents classified as immunotherapy according to the NCDB are listed in their Participant User File (PUF) data dictionary [17].

### Statistical analysis

We compared two groups: patients receiving chemoimmunotherapy versus those receiving chemotherapy alone; baseline clinical and demographic characteristics were compared using χ^2^tests for categorical variables and t-tests for continuous variables.

We performed multivariable Cox-Proportional Hazards regression to assess the association of chemoimmunotherapy treatment and overall survival (OS) according to sex, and adjusting for age, histology, race, comorbidity index, receipt of palliative radiation, insurance status, median household income and year of diagnosis. An interaction term between sex and receipt of chemoimmunotherapy was added to the model and a p _interaction_ < 0.05 was considered statistically significant. Subsequently, propensity scores estimating the likelihood that each patient received immunotherapy were calculated separately for males and females, based on all other covariates. Within the male and female subpopulations, an optimal algorithm was used to create a 1:1 match to create balanced cohorts within each sex, as described by Greene and Stuart [20, 21]. Cox proportional hazards regression, accounting for matching, was run on the combined (male and female) propensity-matched cohort to assess the association of immunotherapy and OS by sex; an interaction term of sex*immunotherapy was added and considered significant at a *p* value < 0.05. As an additional sensitivity analysis, we also stratified both the adjusted and propensity matched analyses by histological type. As a secondary objective, for the subset of patients receiving immunotherapy, we used multivariable Cox proportional hazards regression to assess the independent association of gender with overall survival.

All statistical analysis was performed using SAS version 9.4 (Cary, NC: SAS Institute Inc.). The American College of Surgeons and the Commission on Cancer have not verified and are not responsible for the analytic or statistical methodology used, or the conclusions drawn from these data by the investigator. This study was determined to be human subject research exempt by the institutional review board of Mount Sinai Hospital. All methods were carried out in accordance with relevant guidelines and regulations.

## Results

### Patient characteristics

Of the 12,797 patients that met selection criteria, 10,733 (84%) received chemotherapy alone and 2064 (16%) received chemoimmunotherapy. Those receiving chemoimmunotherapy were significantly more likely to be younger (*p* < 0.0001), have adenocarcinoma (p < 0.0001), be white (p < 0.0001), and have private health insurance (p < 0.0001) (Table 1), than those receiving chemotherapy alone.

**Table 1:** Clinical variables according to chemoimmunotherapy status

	Chemotherapy Alone (*n* = 10,733 (83.9%)) n (%)	Chemoimmunotherapy (*n* = 2064 (16.1%)) n (%)	*p* value
**Age (years)**	66.6 (sd 9.1)	65.1 (sd 8.5)	< 0.0001
**Sex**			0.7613
Male	5707 (53.2)	1105 (53.5)	
Female	5026 (46.8)	959 (46.5)	
**Histology**			< 0.0001
Adenocarcinoma	6858 (63.9)	1648 (79.8)	
Squamous	2146 (20.0)	180 (8.7)	
Large Cell or other	1729 (16.1)	236 (11.4)	
**Race**^a^			0.0583
White	8674 (81.4)	1698 (82.8)	
Black	1390 (13.1)	266 (13.0)	
Asian/Other	588 (5.5)	87 (4.2)	
**Charlson Comorbidity Score**			0.5969
0	7049 (65.7)	1361 (65.9)	
1	2535 (23.6)	497 (24.1)	
≥2	1149 (10.7)	206 (10.0)	
**Palliative Radiation**			0.9970
No	6589 (61.4)	1267 (61.4)	
Yes	4144 (38.6)	797 (38.6)	
**Insurance Status**^b^			< 0.0001
Uninsured	344 (3.3)	55 (2.7)	
Private	3359 (31.9)	781 (38.7)	
Public	6837 (64.9)	1183 (58.6)	
**Area of residence median household income, quartiles ($)** ^c^			0.3918
< 38,000	2085 (19.5)	374 (18.2)	
38,000 ­ 47,999	2634 (24.6)	492 (23.9)	
48,000 ­ 62,999	2804 (26.2)	551 (26.81)	
≥63,000	3184 (29.7)	638 (31.1)	

*sd* standard deviation

^a^missing data for 94 patients

^b^ missing data for 238 patients

^c^ missing data for 35 patients

### Survival

In the full cohort, the median OS those receiving just chemotherapy alone and chemoimmunotherapy was 9.20 and 12.58 months, respectively. Median OS for males and females was and 8.51 and 11.53 months, respectively. Adjusted survival analysis showed that both males (hazard ratio (HR)_adj_: 0.80, 95% CI: 0.74–0.87) and females (HR_adj_: 0.83, 95% CI: 0.76–0.90) had better OS when treated with chemoimmunotherapy, compared to chemotherapy alone. There was no statistically significant interaction between sex and receipt of immunotherapy (p for interaction = 0.63). These findings were consistent in the propensity-score matched analysis (HR: 0.80, 95% CI: 0.72–0.88; HR: 0.88, 95% CI: 0.79–0.99 for males and females, respectively; p for interaction = 0.21) (Table 2).

**Table 2:** Association between chemoimmunotherapy and overall survival, by sex

Multivariable Analysis (*n* = 12,431)	
	**HR**_**adj**_^a^ **(95% CI)**
Chemoimmunotherapy Yes vs. No, Males	0.80 (0.74–0.87)
Chemoimmunotherapy Yes vs. No, Females	0.83 (0.76–0.90)
Immunotherapy*Sex	*p* = 0.6250
Propensity Matched Analysis (*n* = 3814; 1907 pairs)^a^	
	**HR (95% CI)**
Chemoimmunotherapy Yes vs. No, Males	0.80 (0.72–0.88)
Chemoimmunotherapy Yes vs. No, Females	0.88 (0.78–0.99)
Immunotherapy*Sex	*p* = 0.2162

^a^adjusted for/ propensity matched on sex, age at diagnosis, histology (adenocarcinoma, squamous, large cell or other), race (white, Black or Asian/other), Charlson-Deyo comorbidity index, receipt of palliative radiation, year of diagnosis, insurance status (uninsured, privately insured, publically insured, unknown), and median household income for patient’s area of residence (< $38,000, $38,000–$47,999, $48,000–$62,999, >$63,000), * interaction effect

*Abbreviations*: *HR* Hazard Ratio, *CI* Confidence Interval

In the adenocarcinoma subgroup, chemoimmunotherapy treatment was associated with better OS for both males (HR_adj_: 0.82, 95% CI: 0.75–0.90) and females (HR_adj_: 0.85, 95% CI: 0.77–0.94), with no statistically significant interaction between sex and receipt of immunotherapy (p for interaction = 0.65) in the adjusted survival model. These results were consistent after propensity score matching (HR: 0.79, 95% CI: 0.70–0.88; HR: 0.85, 95% CI: 0.74–0.96 for males and females, respectively; p for interaction = 0.43). In patients with squamous histology, males derived more benefit from the addition of immunotherapy to chemotherapy treatment (HR_adj_: 0.73, 95% CI: 0.58–0.91 and HR: 0.86, 95% CI: 0.61–1.21, adjusted and propensity matched models, respectively). Female benefit was not apparent (HR_adj_: 1.03, 95% CI: 0.76–1.38 and HR: 0.97, 95% CI: 0.62–1.53, adjusted and propensity matched). In the adjusted model, the efficacy of chemoimmunotherapy treatment was marginally statically significantly different between male and female patients (p for interaction = 0.07) The p for interaction in the propensity matched model (*n* = 276) was not statistically significant (*p* = 0.67) (Table 3).

**Table 3:** Association between chemoimmunotherapy and overall survival according to histology and sex

Multivariable Analysis		
	**Adenocarcinoma (*n*** **= 8256)**	**Squamous Cell Carcinoma (*n*** **= 2263)**
	**HR**_**adj**_^a^ **(95% CI)**	**HR**_**adj**_^a^ **(95% CI)**
Chemoimmunotherapy Yes vs. No, Males	0.82 (0.75–0.90)	0.73 (0.58–0.91)
Chemoimmunotherapy Yes vs. No, Females	0.85 (0.77–0.94)	1.03 (0.76–1.38)
Immunotherapy*Sex	*p* = 0.6496	*p* = 0.0710
Propensity Matched Analysis^a^		
	**Adenocarcinoma (*n*** **= 2958, 1479 pairs)**	**Squamous Cell Carcinoma** **(*n*** **= 276; 138 pairs)**
	**HR (95% CI)**	**HR (95% CI)**
Chemoimmunotherapy Yes vs. No, Males	0.79 (0.70–088)	0.86 (0.61–1.21)
Chemoimmunotherapy Yes vs. No, Females	0.85 (0.74–0.96)	0.97 (0.62–1.53)
Immunotherapy*Sex	*p* = 0.4347	*p* = 0.6760

^a^adjusted for/propensity matched on sex, age at diagnosis, histology (adenocarcinoma, squamous, large cell or other), race (white, Black or Asian/other), Charlson-Deyo comorbidity index, receipt of palliative radiation, year of diagnosis, insurance status (uninsured, privately insured, publically insured, unknown), and median household income for patient’s area of residence (< $38,000, $38,000–$47,999, $48,000–$62,999, >$63,000), * interaction effect

*Abbreviations*: *HR* Hazard Ratio, *CI* Confidence Interval

Among the subset of patients who received chemoimmunotherapy female sex (HR_adj_ 0.79, 95% CI: 0.71–0.88), was associated with better OS. After propensity matching to balance the cohort across sex, females had significantly better OS than males (HR 0.84, 95% CI: 0.72–0.97; Table 4).

**Table 4:** Survival analysis in NSCLC patients treated with chemoimmunotherapy

Immunotherapy Patients		
	Multivariable Analysis^a^ (***n*** = 1998)	Propensity Matched Analysis (***n*** = 1072; 536 pairs)^a^
	**HR**_**adj**_ **(95% CI)**	**HR (95% CI)**
**Sex**: Female/Male	0.79 (0.71–0.88)	0.84 (0.72–0.97)

^a^adjusted for/propensity matched on sex, age at diagnosis, histology (adenocarcinoma, squamous, large cell or other), race (White, Black or Asian/other), Charlson-Deyo comorbidity index, receipt of palliative radiation, year of diagnosis, insurance status (uninsured, privately insured, publically insured, unknown), and median household income for patient’s area of residence (< $38,000, $38,000–$47,999, $48,000–$62,999, >$63,000)

*Abbreviations*: *HR* Hazard Ratio, *CI* Confidence Interval

## Discussion

In this study, both males and female stage IV NSCLC patients treated with chemoimmunotherapy were found to have better overall survival compared to those treated with chemotherapy alone. In the overall patient cohort, benefit from the addition of immunotherapy was similar between sexes. However, when stratifying by the main histological subtypes of NSCLC, we did not observe any difference in immunotherapy efficacy between male and female adenocarcinoma patients, although our results preliminarily suggest a greater degree of benefit in male versus female squamous cell patients. Lung adenocarcinoma and squamous cell carcinoma, while subtypes of the same disease, are in fact known to vary dramatically both in terms of their genomic and clinical attributes [22]. More recently, key differences between these histological subtypes has been observed in terms of immune host response, with implications for immunotherapy response [22]. Sample size of this sub-group was small, however, especially in the propensity-matched cohort; replication of this finding in a much larger cohort of squamous cell patients is necessary.

In meta-analyses conducted by Conforti et al., they found that males derive significantly more clinical benefit than females when treated with immune checkpoint inhibitors for NSCLC, yet found the opposite to be true when studying the effects of the addition of immunotherapy to chemotherapy, reporting pooled survival estimates of 0.76 (95% CI = 0.66 to 0.87) and 0.48 (95% CI = 0.35 to 0.67) for men and women, respectively [12, 14]. By comparison, our analysis showed the addition of immunotherapy treatment as having a smaller impact on survival, and we did not find any evidence of female NSCLC patients deriving more benefit from chemoimmunotherapy. Estimates from Conforti et al. are based on results of randomized clinical trials, the patients of which are traditionally known to differ both clinically and demographically from patients in real-world settings. The authors also acknowledge as a major limitation the use of data extracted from published studies, which precluded them from being able to adjust for patient-level characteristics that differ by sex and may affect treatment outcomes. Our study used population-based, patient-level data that included key clinical and demographic information that allowed for adjustment for potential confounders, such as age, comorbidity, and income, using several different statistical modeling techniques. Consideration of these confounders is critical when investigating the impact of sex on immunotherapy response; we show here the tremendous importance of taking into account histological subtype, which isn’t possible in most meta-analyses. Moreover, even when clinical trials are stratified by histological subtype, such as the KEYNOTE-407 trial investigating pembrolizumab plus chemotherapy in patients with metastatic squamous NSCLC, female patients are often dramatically underrepresented [23]. In this recent trial, just 19% of study participants were female [23]. While a limitation of our analysis is that only 2015 data was available, with FDA-approval of immunotherapy treatment for NSCLC beginning later in that year, the high percentage (47%) of chemoimmunotherapy patients being female attests to the enrichment of real-world patients over RCT participants in our data. Using real-world, patient-level data is necessary is necessary to more fully understand disparities in chemotherapy plus immunotherapy response, and we hope to see this work replicated in larger, more recent datasets in future.

NCDB is in fact one of the first large clinical datasets to include data for immunotherapy treatment along with long-term survival data. A significant limitation of immunotherapy trials is the lack of long-term follow up, resulting in the use of surrogate endpoints such as response rates and progression free survival to estimate benefit, [24] which may not be entirely representative of actual benefit [25]. We consider it a notable strength of our study that NCDB has highly accurate mortality data that spans multiple years on a large sample size, creating an opportunity to assess the true benefits of immunotherapy in specific patient subsets.

While our results did benefit from the comprehensiveness of the NCDB data, there were important variables missing from the data, the exclusion of which may be influencing the results. Specifically, we did not have key elements such as ECOG status; if patients with lower ECOG status were less likely to receive immunotherapy, the results could be biased towards showing the combination of immunotherapy + chemotherapy to be more efficacious than it actually is. We were able, however, to adjust for comorbidity status which should be a good proxy for EGOG performance. In addition, patients’ sex is not known to be associated with ECOG status, [26] thus ECOG should not have affected the observed relative difference in treatment efficacy between the sexes. Because the NCDB is a clinical database sourced from registry data, other potential confounders, such as EGFR, ALK, ROS mutational status, which are known predictive biomarkers for NSCLC, and differ by sex, were missing in the NCDB. Since women are more likely to harbor these mutations [27] the inability to adjust for these markers is a considerable limitation. However, the incidence of driver mutations in squamous cell lung cancers is extremely low, [28] therefore EGFR/ALK/ROS1 mutations should have no impact on the findings reported for the stratified analysis on squamous cell lung cancer. Smoking status, too, was missing from the NCDB data. While all histologic types of lung cancer are significantly associated with cigarette smoking, this association is strongest for squamous cell carcinoma [29]. Additionally, smoking history is more commonly reported for male patients than female. Stratifying according to histology and sex should reduce some bias, but we acknowledge that smoking status is an important potential confounder we could not fully adjust for. We were also unable to study the role of tumor mutation burden and the tumor microenvironment, which may be impacted by smoking [30]. While the results reported here of our analysis using individual patients’ data are meant to overcome some of the limitations of the previous meta-analyses conducted on this topic, future research is needed to explore how some of the covariates lacking in the NCDB might influence immunotherapy response according to sex. As larger, more detailed datasets containing immunotherapy information become available, we recommend that this analysis be repeated. Use of electronic health registry data, for example, might be one avenue to overcome these limitations.

Another major limitation to this work is that the specific type of immunotherapy agent used was not reported in the NCDB. Moreover, several of the drugs included in the NCDB immunotherapy variable are not FDA-approved or conventional immunotherapies, although all agents included have been shown in preclinical and/or clinical studies to modulate the anti-tumor immune response. Never-the-less, we have reason to believe that this patient population is enriched for those who did in fact receive immune checkpoint inhibitors. The patient selection helped us to reduce the heterogeneity of drugs included under the “immunotherapy” variable in the NCDB. By limiting the analysis to only those patients diagnosed in 2015, the year the FDA approved the first immunotherapy drugs for NSCLC treatment, we have enriched for patients receiving immune checkpoint inhibition drugs compared to patients diagnosed in previous years. We also tried to get an estimate of which types of drugs would have been available for clinical use in 2015 and onward, and how likely it is that patients included in the present analysis were treated with immunotherapeutic agents that are now excluded from clinical use. We observed that many of the immunotherapeutic treatments included as part of the NCBD’s immunotherapy variable have never been approved for use among NSCLC patients, making it unlikely that our patient population were treated with these. For instance, the NCDB immunotherapy variable is inclusive of EGFR antibodies, but these drugs were never FDA approved for standard of care therapy in NSCLC, so it is doubtful that the patients in our dataset received this form of treatment. Moreover, we completed an extensive search of the literature, including clinicaltrails.gov and PubMed, looking for clinical trials using EGFR antibody treatment for NSCLC during our study period [31]. All trials inclusive of EGFR antibodies were conducted only in combination with chemotherapy treatment. We therefore conduced a sensitivity analysis on immunotherapy only vs chemotherapy only treatment, and found that there was no statistically significant interaction between sex and immunotherapy efficacy (*p* > 0.05, data not shown). Stratification according to histologic type should have also helped to provide a cleaner picture of immunotherapy efficacy by sex. In squamous cell carcinoma cases angiogenesis inhibitors are contraindicated due to increased risk of bleeding, [16] thus we expect that the squamous cell lung cancer patients recorded as treated with immunotherapy would not have received anti-VEGF treatment. In this stratified analysis we observed a difference in treatment efficacy between sexes when immunotherapy was added to chemotherapy. Ideally this analysis will be replicated in a larger patient-level dataset reporting specific immune checkpoint inhibitor information; it would be interesting to see if the difference in efficacy observed among squamous cell patients is seen among those with adenocarcinoma when immunotherapy drugs are limited concisely to immune checkpoint inhibitors. The lack of details on type of immunotherapy remains a notable limitation of our work, yet we believe that our findings contribute to the general understanding of the association of sex and immunotherapy, and raise important questions for future research.

The role of sex as it pertains to immunotherapy response remains an important clinical question. The patient-level nature of our data allowed us to also do a head-to-head comparison of survival outcomes of females vs males receiving immunotherapy in addition to chemotherapy. We found that females in fact experienced improved survival with immunotherapy compared to males, after adjusting for potentially important clinical variables. This finding may be expected given that females experience better cancer outcomes overall than males [32]. Lung cancers of male and females are known to have distinctive clinical and biological differences, notably in terms of histology, driver mutations and smoking exposure history, [33, 34] all of which may impact immunotherapy efficacy. We would like to note, however, that the major aim this work is not demonstrate the value of chemoimmunotherapy treatment, generally or for specific subgroups, but instead to investigate how chemoimmunotherapy treatment efficacy may vary by sex and/or histology.

## Conclusion

Immune checkpoint inhibitors and other immunotherapeutical approaches have revolutionized cancer treatment, and understanding which patients will be most likely to benefit from these treatments remains crucial to improving lung cancer outcomes. The study adds new data and should be regarded as an important contribution to the ongoing discussion on sex and immunotherapy treatment in NSCLC. How histology impacts sex*immunotherapy efficacy is a subject warranting future research.

The biological mechanisms whereby sex may impact immunotherapy benefit is an important scientific question, but one that may not be answerable when looking at NSCLC as a whole, and instead may require more specific subtype analysis in real-world, patient-level datasets.

## Supplementary Information

**Additional file 1: Supplemental Fig. 1.** Patient Selection. **Supplemental Table 1.** Drug agents classified as immunotherapy according to the NCDB.

## Data Availability

National Cancer Database (NCDB) data is available at no cost to members of the American College of Surgeons through an application process. A public access database may be made available in the near future. For more information: https://www.sgim.org/communities/research/dataset-compendium/national-cancer-database-ncdb. Views expressed in this article do not communicate an official position of the NIH nor the American College of Surgeons.
