# Supplementary material for: Modulation of chemoimmunotherapy efficacy in non-small cell lung cancer by sex and histology: a real-world, patient-level analysis
Source: BMC Cancer. 2022 Jan 19;22:80. doi: 10.1186/s12885-022-09187-y (PMC8767728; doi:10.1186/s12885-022-09187-y)
Supplement: Supplementary file 1 — Additional file 1: Supplemental Fig. 1. Patient Selection. Supplemental Table 1. Drug agents classified as immunotherapy according to the NCDB. [file 12885_2022_9187_MOESM1_ESM.docx]

Supplemental Figure 1: Patient Selection


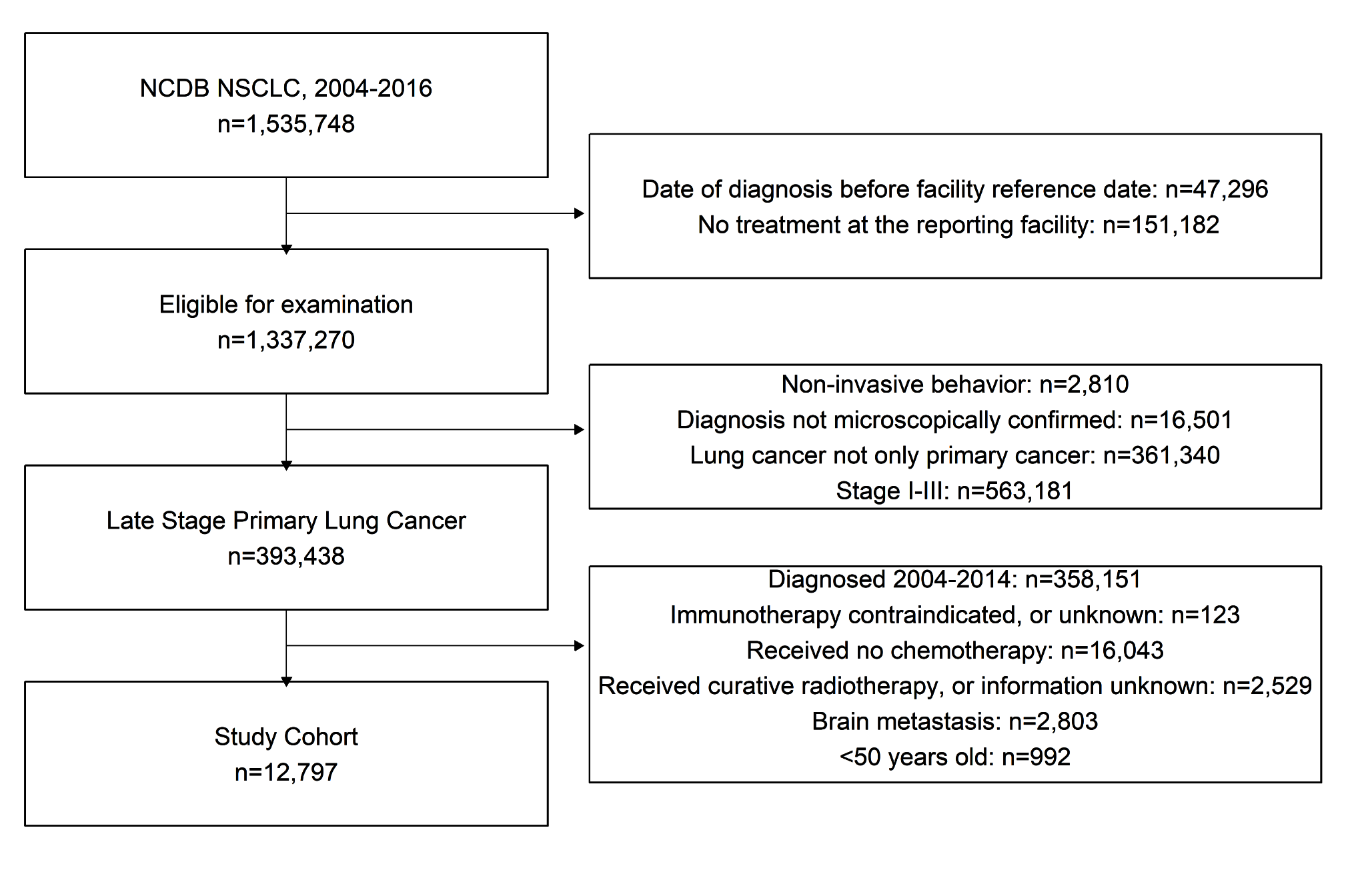


Supplemental Table 1: Drug agents classified as immunotherapy according to the NCDB

| Checkpoint Inhibitors | Atezolizumab  Durvalumab  Ipilimumab  Nivolumab  Pembrolizumab |
| --- | --- |
| Anti-VEGF/ Angiogenesis inhibitor | Bevacizumab  Cyramza |
| EGFR Antibody | Cetuximab  Dalotuzumab  Necitumumab  Panitumumab |
| Vaccines/ Adenoviral p53 Gene Therapy | BLP25  BMS-936558  CEA-Vac  EC-2101  Dendritic cell-P53  Egf Cancer Vaccine  Fowlpox-CEA (6D)  GVAX Lung Cancer Vaccine  INGN 201  IV IL-2 Gene Medicine  Lung Cancer Vaccine  MGV Vaccine  MVA-Muc1-IL2  P53 and RAS vaccine  RAS 5-17 Peptide Vaccine  SB249553  TBC-CEA  Vaccinia-CEA |
| Other | Thalidomide  Matrix Metalloproteinease Inhibitor  Advexin  Interleukin-12  ProMune  RC-3095  Trastuzumab  TriAb  TriGem  recMAGE-A3+AS15 |

* these agents are coded as approved for treatment of lung cancer, in clinical trial for lung cancer, or if lung is designated as a primary site of treatment according to the NCDB. Patients should be coded as yes to receiving immunotherapy (variable name RX_SUMM_IMMUNOTHERAPY) if they have been administered any of these agents variable.
